# Robot Evaluation and Selection with Entropy-Based Combination Weighting and Cloud TODIM Approach

**DOI:** 10.3390/e20050349

**Published:** 2018-05-07

**Authors:** Jing-Jing Wang, Zhong-Hua Miao, Feng-Bao Cui, Hu-Chen Liu

**Affiliations:** 1 School of Management, Shanghai University, Shanghai 200444, China; 2 School of Mechatronic Engineering and Automation, Shanghai University, Shanghai 200444, China; 3 School of Economics and Management, Tongji University, Shanghai 200092, China; 4 Department of Economics & Management, Yibin University, Yibin 644007, China

**Keywords:** robot selection, cloud model, TODIM method, combination weight, entropy method

## Abstract

Nowadays robots have been commonly adopted in various manufacturing industries to improve product quality and productivity. The selection of the best robot to suit a specific production setting is a difficult decision making task for manufacturers because of the increase in complexity and number of robot systems. In this paper, we explore two key issues of robot evaluation and selection: the representation of decision makers’ diversified assessments and the determination of the ranking of available robots. Specifically, a decision support model which utilizes cloud model and TODIM (an acronym in Portuguese of interactive and multiple criteria decision making) method is developed for the purpose of handling robot selection problems with hesitant linguistic information. Besides, we use an entropy-based combination weighting technique to estimate the weights of evaluation criteria. Finally, we illustrate the proposed cloud TODIM approach with a robot selection example for an automobile manufacturer, and further validate its effectiveness and benefits via a comparative analysis. The results show that the proposed robot selection model has some unique advantages, which is more realistic and flexible for robot selection under a complex and uncertain environment.

## 1. Introduction

Due to the explosion of information technology and engineering sciences, robots have been widely utilized in many manufacturing practices. An industry robot is defined as a self-controlled, reprogrammable, and multi-functional machine made of mechanical, microelectronic, and electrical components [1,2,3]. Robots are capable of executing monotonous, complicated, and hazardous tasks with precision, and thus used by manufacturers in many applications, including assembly, material handling, spray painting, and packaging [4,5]. Proper evaluation of robots and selecting the most appropriate one for a particular production environment would be helpful for a company to improve product quality and build profitability. Because of the increasing complexity of robot systems and the growing number of options on the market with different capabilities, features, and specifications, selecting the most suited robotic system for a particular industrial application is not an easy task for production firms. Therefore, in recent years, the problem of robot selection has attracted extensive attention from researchers as well as practitioners [6,7,8].

Normally, robot selection is accomplished by multiple decision makers from different technical expertise and working backgrounds. In practice, it is hard for decision makers to give their opinions about alternatives using numerical values. Instead, they often use linguistic terms to express the assessments for alternate robots versus different criteria and criteria weights. As a new cognition model, cloud model was suggested by Li et al. [9] based on probability statistics and fuzzy sets for handle fuzziness, randomness, and uncertain concepts. The prominent feature of the cloud model is that it can perfectly deal with the uncertainty of qualitative notions and make the bidirectional conversion between qualitative concepts and quantitative information more easily [10,11,12]. Recently, researchers have applied cloud model theory to address linguistic decision making problems in many areas. For example, Wu et al. [11] proposed a modified multi-objective optimization by ratio analysis plus the full multiplicative form (MULTIMOORA) method based on cloud model theory for quality function deployment analysis. Wang et al. [12] reported an extended QUALIFLEX (qualitative flexible multiple criteria) method with cloud model theory to assess the performance of suppliers under economic and environmental criteria. Liu and Wen [13] proposed a cloud model-based robust algorithm for continuum topology optimization considering uncertainties in load locations. Wu et al. [14] designed an applicable method using cloud model with 2-order additive fuzzy measures for the selection of waste-to-energy disposal sites in China. Wang et al. [15] suggested a consensus-based method based on cloud model for the large group decision making with linguistic information. In addition, Liu et al. [16] used a grey relational analysis method and cloud model to evaluate failure modes with incomplete weight information of risk factors. Chang and Wang [17] employed cloud model and decision tree to assess teacher evaluation in higher education including subjectivity, inaccuracy, and fuzziness.

On the other hand, various factors, such as positioning accuracy, cost, programming flexibility, load capacity, man-machine interface, and vendor’s service quality, need to be considered in the robot selection decision making [7,18,19]. Therefore, robot selection is basically a multiple criteria decision making (MCDM) problem [3,8], and organizations can find utility in MCDM methods that can assist with evaluating and selecting robots in view of different conflict criteria. The TODIM (an acronym in Portuguese of interactive and multiple criteria decision making) [20] is an MCDM method on the basis of prospect theory, which uses prospect formula to determine the supremacy of one alternative against another and considers experts’ bounded rationality in the decision making processes. Due to its advantages, the TODIM method has been applied by many researchers in different decision making solutions. For instance, Zhang and Xu [21] suggested a hesitant vague TODIM approach for the evaluation of sustainable water management efficiency. Wang et al. [22] developed a likelihood-based TODIM method based on multi-hesitant fuzzy linguistic sets to select service providers in logistics outsourcing. Sang and Liu [23] proposed an interval type-2 fuzzy set-based TODIM approach to address multiple criteria green supplier selection problems. Ji et al. [24] reported a projection-based TODIM model to deal with personnel selection problems within the multi-valued neutrosophic environment. In [25], an extended TODIM model was presented for the evaluation of mineral resources development efficiency with hesitant fuzzy linguistic information. In [26], an intuitionistic linguistic TODIM method was proposed to deal with the interactive MCDM problems in which criteria weights are unknown. Besides, Hu et al. [27] addressed the online diagnosis and medical treatment selection problems by using a TODIM-based three-way decision model, and Wang et al. [28] managed the non-homogeneous information and experts’ psychological behavior in group emergency decision making by using a fuzzy TODIM method.

Against the above background, this article aims to develop a cloud model based-TODIM (cloud TODIM) approach to handle robot selecting problems within an uncertain linguistic context. The proposed decision making approach brings several contributions to robot evaluation and selection. First, various linguistic assessments of decision makers on alternative robots are described with the aid of cloud model theory. Second, a new type of the standard TODIM is developed for determining the best robot for a given industrial application problem. Third, a combination weighting method is utilized to indicate criteria weights, which considers both subjective and objective weights in the robot selection process. In addition, a real example is presented to display the feasibility and effectiveness of the proposed cloud TODIM method for selecting robots.

The rest of this article is arranged as below: Section 2 briefly reviews current robot selection methods in the literature. Section 3 presents the basic concepts and operations regarding cloud model theory. In Section 4, we give a new robot evaluation and selection framework by combing cloud model theory with the TODIM method. In Section 5, we exemplify the proposed cloud model-TODIM methodology by using a practical robot selection case and by comparing with some existing methods. Finally, the conclusions of this paper and future research suggestions are provided in Section 6.

## 2. Literature Review

In the past decades, a wide variety of methods and tools have been proposed for supporting robot selection decision making in different industrial contexts. Generally, the approaches of robot assessment and selection can be categorized into four categories: MCDM methods, optimization models, computer aided methods, and other solutions (e.g., statistical and mathematical models) [2,29]. Due to the multi-criteria character of robot selection problems, the MCDM methods have been recognized as a promising tool which reduces the problem of human’s decision impact on the final result of selection process. Thus, in the following, we mainly review the MCDM-based models suggested in previous studies for supporting the robot selection process. For a comprehensive review of more robot selection methods, one can refer to [29].

Xue et al. [8] developed a linguistic MCDM model, which integrates hesitant linguistic term sets with an improved QUALIFLEX technique, for robot selection with partial criteria weight information. Sen et al. [3] presented an extension of the preference ranking organization method for enrichment evaluation (PROMETHEE) for robot evaluation considering objective and subjective data simultaneously, and Sen et al. [7] applied the PROMETHEE II method for addressing the robot selection problem subjected to a set of objective data. An extended VIKOR approach was developed by Keshavarz Ghorabaee [6] for robot evaluation under the interval type-2 fuzzy environment, and Kavita [30] suggested an improved VIKOR method for the evaluation of robots in the context of triangular intuitionistic vague sets. Gitinavard et al. [4] presented an interval hesitant fuzzy COPRAS (COmplex Proportional ASsessment of alternatives) model and Vahdani et al. [31] offered an interval-valued fuzzy COPRAS framework for the determination of the most appropriate robot under uncertainty. Durán and Aguilo [32] developed a fuzzy analytic hierarchy process (AHP)-based decision support system for selecting machine tools, and Kumru and Kumru [33] used a fuzzy analytic network process (ANP) model for the selection of 3D coordinate-measuring machines. Besides, a number of other models have been suggested to help experts in their robot selection decisions, such as the generalized interval-valued fuzzy TOPSIS [34], the fuzzy hierarchical TOPSIS [5], the weighted aggregated sum product assessment (WASPAS) [35,36], and the ELECTRE (Elimination and Choice Expressing the Reality) method [37].

In Koulouriotis and Ketipi [38], a fuzzy digraph methodology was proposed for the comparison and selection of industrial robots, in which the fuzzy logic was used for managing experts’ assessments and the digraph and matrix technique was adopted for ranking robots. Gola and Świć [39] proposed a computer aided machine tool selection tool for focused flexibility manufacturing systems considering several economical criteria. Rao et al. [1] proposed a MCDM technique to evaluate and rank robots in an industry application, which utilizes statistical variance and AHP method to compute the objective and subjective weights of criteria, respectively. Kentli and Kar [40] presented a multi-criteria robot selection algorithm on the basis of satisfaction function and distance measure, and Kumar and Garg [41] gave a determining quantitative framework using the distance method to choose the optimal robot. In addition, multiple MCDM methods have been used for industrial robot selection. For instance, Parameshwaran et al. [19] proposed a united approach for the assessment of robots from objective and subjective standards, in which fuzzy AHP was utilized to indicate criteria weights and fuzzy TOPSIS and fuzzy VIKOR were employed to determine rank orders of alternatives. Chatterjee et al. [2] utilized the VIKOR and ELECTRE methods for the selection of industrial robots, and Bairagi et al. [42] presented a de novo multi-approaches multi-criteria decision making method called Technique of Precise Order Preference (TPOP) for the performance evaluation of material handling devices.

The literature review above shows that a lot of MCDM methods have been applied for robot selection decision making. However, the psychological behavior of experts has not been taken into consideration in the existing models of robot selection, which is of considerable significance since different experts may have different psychological expectations in the practical situation. On the other hand, although many uncertainty theories, such as fuzzy sets, intuitionistic vague sets and 2-tuple linguistic variables, have been utilized by researchers for coping with imprecise and fuzzy decision makers’ preferences, they cannot describe the fuzziness and randomness of qualitative characteristics of criteria simultaneously. Therefore, to bridge theses gaps, this paper attempts to developed a novel behavioral decision making model to address the problem of robot evaluation and selection with hesitant linguistic information. Furthermore, both subjective and objective criteria weights are taken into account in determining the ranking orders of alternative robots. Finally, we apply the proposed cloud TODIM approach to the robot selection case in an auto company to demonstrate its validity and applicability.

## 3. Basic Concepts

### 3.1. Cloud Model Theory

**Definition** **1** [9,10]**.** *Supposing a qualitative concept T defined on a universe of discourse U, let x(x∈U) be a random instantiation of the concept T and y∈[0,1] be the certainty degree of x belonging to T, which corresponds to a random number with a stable tendency. Then the distribution of x in the universe U is called a cloud, and the cloud drop is denoted as (x, y).*

**Definition** **2** [9,10]**.** *The characteristics of a cloud y are depicted by expectation Ex, entropy En, and hyper entropy He. Here, Ex is the center value of the qualitative concept domain, En measures the randomness and fuzziness of the qualitative concept, and He reflects the dispersion of the cloud drops and the uncertain degree of the membership function. Based on the three numerical characteristics, a cloud can be described as $\tilde{y}=\left(Ex,En,He\right)$.*

Generally, different kinds of clouds exist in the literature, such as normal cloud, trapezium cloud, triangular cloud, and half cloud. Among them, the normal cloud based on normal distribution and Gaussian membership is an important cloud model widely applied by researchers.

**Definition** **3.** *Considering any two normal clouds ${\tilde{y}}_{1}=\left(Ex_{1},En_{1},He_{1}\right)$ and ${\tilde{y}}_{2}=\left(Ex_{2},En_{2},He_{2}\right)$ in the universe U, some basic operations between them are defined as follows [14,43]:*

(1) ${\tilde{y}}_{1}+{\tilde{y}}_{2}=\left(Ex_{1}+Ex_{2},\sqrt{En_{1}^{2}+En_{2}^{2}},\sqrt{He_{1}^{2}+He_{2}^{2}}\right);$
(2) ${\tilde{y}}_{1}\times {\tilde{y}}_{2}=\left(Ex_{1}Ex_{2},\sqrt{{\left(En_{1}Ex_{2}\right)}^{2}+{\left(En_{2}Ex_{1}\right)}^{2}},\sqrt{{\left(He_{1}Ex_{2}\right)}^{2}+{\left(He_{2}Ex_{1}\right)}^{2}}\right);$
(3) $\lambda {\tilde{y}}_{1}^{}=\left(\lambda Ex_{1},\sqrt{\lambda }En_{1},\sqrt{\lambda }He_{1}\right),\;\;\;\lambda >0;$
(4) ${\tilde{y}}_{1}^{\lambda }=\left(Ex_{1}^{\lambda },\sqrt{\lambda }{\left(Ex_{1}\right)}^{\lambda -1}En_{1},\sqrt{\lambda }{\left(Ex_{1}\right)}^{\lambda -1}He_{1}\right),\;\;\;\lambda >0$.

**Definition** **4.** *Let ${\tilde{y}}_{i}=\left(Ex_{i},En_{i},He_{i}\right)\left(i=1,2,\ldots ,n\right)$ be a collection of normal clouds in the universe U, and $w={\left(w_{1},w_{2},\ldots ,w_{n}\right)}^{T}$ be their associated weights with $w_{i}\in \left[0,1\right]$ and $\sum_{i=1}^{n}w_{i}=1$. Then the cloud weighted averaging (CWA) is defined as [43]:*

(1) $${\mathrm{CWA}}_{w}\left({\tilde{y}}_{1},{\tilde{y}}_{2},\ldots ,{\tilde{y}}_{n}\right)=\sum_{i=1}^{n}w_{i}{\tilde{y}}_{i}=\left(\sum_{i=1}^{n}w_{i}Ex_{i},\sqrt{\sum_{i=1}^{n}w_{i}En_{i}^{2}},\sqrt{\sum_{i=1}^{n}w_{i}He_{i}^{2}}\right).$$

**Definition** **5.** *Assume that there are two normal clouds ${\tilde{y}}_{1}=\left(Ex_{1},En_{1},He_{1}\right)$ and ${\tilde{y}}_{2}=\left(Ex_{2},En_{2},He_{2}\right)$ in the universe U, the distance between them is defined as [44]:*

(2) $$\begin{array}{l} d\left({\tilde{y}}_{1},{\tilde{y}}_{2}\right)=|\left(1-\frac{{\left(En_{1}\right)}^{2}+{\left(He_{1}\right)}^{2}}{{\left(En_{1}\right)}^{2}+{\left(He_{1}\right)}^{2}+{\left(En_{2}\right)}^{2}+{\left(He_{2}\right)}^{2}}\right)Ex_{1}-\\ \;\;\;\;\;\;\;\;\;\;\;\;\;\;\;\;\;\left(1-\frac{{\left(En_{2}\right)}^{2}+{\left(He_{2}\right)}^{2}}{{\left(En_{1}\right)}^{2}+{\left(He_{1}\right)}^{2}+{\left(En_{2}\right)}^{2}+{\left(He_{2}\right)}^{2}}\right)Ex_{2}|. \end{array}$$

Especially, when $En_{1}=He_{1}=En_{2}=He_{2}=0$, the cloud distance changes to be the distance between crisp numbers and $d\left({\tilde{y}}_{1},{\tilde{y}}_{2}\right)=\left|Ex_{1}-Ex_{2}\right|$.

**Definition** **6.** *Let ${\tilde{y}}_{i}=\left(Ex_{i},En_{i},He_{i}\right)\left(i=1,2,\ldots ,n\right)$ be a collection of normal clouds in the universe U, and $\omega =\left(\omega_{1},\omega_{2},\ldots ,\omega_{n}\right)$ be an associated weight vector satisfying $\omega_{j}\in \left[0,1\right]$ and $\sum_{j=1}^{n}\omega_{j}=1$, then the cloud ordered weighted averaging (COWA) is defined as [43]:*

(3) $$\begin{array}{c} {\mathrm{COWA}}_{\omega }\left({\tilde{y}}_{1},{\tilde{y}}_{2},\ldots ,{\tilde{y}}_{n}\right)=\sum_{j=1}^{n}\omega_{j}{\tilde{y}}_{\sigma \left(j\right)}\\ \;\;\;\;\;\;\;\;\;\;\;\;\;\;\;\;\;\;\;\;\;\;=\left(\sum_{j=1}^{n}\omega_{j}Ex_{\sigma \left(j\right)},\sqrt{\sum_{j=1}^{n}\omega_{j}En_{\sigma \left(j\right)}^{2}},\sqrt{\sum_{j=1}^{n}\omega_{j}He_{\sigma \left(j\right)}^{2}}\right), \end{array}$$

where ${\tilde{y}}_{\sigma \left(j\right)}=\left(Ex_{\sigma \left(j\right)},En_{\sigma \left(j\right)},He_{\sigma \left(j\right)}\right)$ is the *j*th largest element of ${\tilde{y}}_{i}\left(i=1,2,\ldots ,n\right)$.

Note that many methods have been proposed in the literature to determine the weights of ordered weighted average (OWA) operator [45], which can also be applied for the COWA operator.

**Definition** **7** [43]**.** *Let ${\tilde{y}}_{i}=\left(Ex_{i},En_{i},He_{i}\right)\left(i=1,2,\ldots ,n\right)$ be a collection of normal clouds in the universe U, and $\omega =\left(\omega_{1},\omega_{2},\ldots ,\omega_{n}\right)$ be an associated weight vector with $\omega_{j}\in \left[0,1\right],and\;\sum_{j=1}^{n}\omega_{j}=1$, then the cloud hybrid averaging (CHA) operator is defined as:*

(4) $$\begin{array}{c} {\mathrm{CHA}}_{\omega ,w}\left({\tilde{y}}_{1},{\tilde{y}}_{2},\ldots ,{\tilde{y}}_{n}\right)=\sum_{j=1}^{n}\omega_{j}{\dot{\tilde{y}}}_{\sigma \left(j\right)}\\ \;\;\;\;\;\;\;\;\;\;\;\;\;\;\;\;\;\;\;=\left(\sum_{j=1}^{n}\omega_{j}\dot{E}x_{\sigma \left(j\right)},\sqrt{\sum_{j=1}^{n}\omega_{j}\dot{E}n_{\sigma \left(j\right)}^{2}},\sqrt{\sum_{j=1}^{n}\omega_{j}\dot{H}e_{\sigma \left(j\right)}^{2}}\right), \end{array}$$

where ${\dot{\tilde{y}}}_{\sigma \left(j\right)}$ is the *j*th largest element of the weighted normal clouds ${\dot{\tilde{y}}}_{i}\left({\dot{\tilde{y}}}_{i}=nw_{i}{\tilde{y}}_{i},i=1,2,\ldots ,n\right),$ $w=\left(w_{1},w_{2},\ldots ,w_{n}\right)$ is the weights of ${\tilde{y}}_{i}\left(i=1,2,\ldots ,n\right)$, with $w_{i}\in \left[0,1\right],$ $\sum_{i=1}^{n}w_{i}=1$, and *n* is the balancing coefficient. Especially, if ω=(1/n,1/n,…,1/n), then the CHA operator reduces to the CWA operator, and if w=(1/n,1/n,…,1/n), then the CHA operator becomes the COWA operator.

### 3.2. Conversion between Linguistic Terms and Clouds

The concept of linguistic variables [46] was proposed to deal with situations which are too complex or too ill-defined to be reasonably represented by quantitative expressions.

**Definition** **8.** *Let $S=\left\{s_{0},s_{1},\ldots ,s_{g}\right\}$ be a finite and totally ordered discrete linguistic term set, where g is an even number and $s_{i}$ denotes a possible value of a linguistic variable, then the linguistic term set has the following characteristics [12,47]:*

(1) Negation operator: Neg (*s_i_*) = *s_j_* such that j=g−i;
(2) The set is ordered: *s_i_* > *s_j_*, if *i* > *j*;
(3) Max operator: max (*s_i_*, *s_j_*) = *s_i_*, if $s_{i}\geq s_{j}$.

**Definition** **9.** Let $S=\left\{s_{0},s_{1},\ldots ,s_{g}\right\}$ be a linguistic term set and [X_min_, X_max_] is an effective domain, then g + 1 basic clouds can be generated corresponding to the linguistic values $s_{i}\left(i=0,1,\ldots ,g\right)$, which are denoted as ${\tilde{y}}_{0}=\left(Ex_{0},En_{0},He_{0}\right),$${\tilde{y}}_{1}=\left(Ex_{1},En_{1},He_{1}\right),\ldots ,$${\tilde{y}}_{g}=\left(Ex_{g},En_{g},He_{g}\right)$, respectively.

Let the linguistic term set $S=\left\{s_{0},s_{1},\ldots ,s_{6}\right\}$, where the valid universe is [*X*_min_, *X*_max_]. By applying a golden radio method, seven basic clouds can be produced and their numerical characteristics are shown below:

$$\begin{array}{l} Ex_{3}=\left(X_{\min}+X_{\max}\right)/2,\;\;Ex_{0}=X_{\min},Ex_{6}=X_{\max},\\ Ex_{2}=Ex_{3}-0.382\left(X_{\max}-X_{\min}\right)/4,Ex_{4}=Ex_{3}+0.382\left(X_{\max}-X_{\min}\right)/4,\\ Ex_{1}=Ex_{3}-\left(X_{\max}-X_{\min}\right)/4,Ex_{5}=Ex_{3}+\left(X_{\max}-X_{\min}\right)/4,\\ En_{2}=En_{4}=0.382\left(X_{\max}-X_{\min}\right)/12,En_{3}=0.618En_{2},\\ En_{1}=En_{5}=En_{2}/0.618,En_{0}=En_{6}=En_{1}/0.618,\\ He_{2}=He_{4}=He_{3}/0.618,He_{1}=He_{5}=He_{2}/0.618,He_{0}=He_{6}=He_{1}/0.618, \end{array}$$

where *He*_3_ can be designated in advance by decision makers.

For example, given the universe [0, 10] and *He*_3_ = 0.1, six basic clouds can be computed as below for the linguistic term set *S* [44]:

$$\begin{array}{l} {\tilde{y}}_{0}=\left(0,0.833,0.424\right),{\tilde{y}}_{1}=\left(2.5,0.515,0.262\right),{\tilde{y}}_{2}=\left(4.05,0.318,0.162\right),\\ {\tilde{y}}_{3}=\left(5,0.197,0.100\right),{\tilde{y}}_{4}=\left(5.96,0.318,0.162\right),{\tilde{y}}_{5}=\left(7.5,0.515,0.262\right),{\tilde{y}}_{6}=\left(10,0.833,0.424\right). \end{array}$$

To depict the hesitancy, fuzziness, and uncertainty of decision makers, Meng et al. [48] defined the linguistic hesitant fuzzy sets (LHFSs) by using linguistic terms and a related membership degree.

**Definition** **10.** Let $S=\left\{s_{0},s_{1},\ldots ,s_{g}\right\}$ be a linguistic term set, a LHFS in S is a set that, when applied to the linguistic terms of S, returns a subset of S with several values in [0, 1], denoted by $LH=\left\{s_{i},lh\left(s_{i}\right)|s_{i}\in S\right\}$, where $lh\left(s_{i}\right)=\left\{r_{1},r_{2},\ldots ,r_{m_{i}}\right\}$ is a set with m_i_ values in [0, 1] denoting the possible membership degrees of the element $s_{i}\in S$ to the set LH.

**Definition** **11** [44]**.** *Let $S=\left\{s_{0},s_{1},\ldots ,s_{g}\right\}$ be a linguistic term set and [X_min_, X_max_] is an effective domain, the corresponding cloud ${\tilde{y}}_{LH}=\left(Ex_{LH},En_{LH},He_{LH}\right)$ of the LHFS $LH=\left\{s_{i},lh\left(s_{i}\right)|s_{i}\in S\right\}$ can be computed by*

(5) $$\begin{array}{l} Ex_{LH}=\frac{1}{\left|\mathrm{index}\left(LH\right)\right|}\left(\sum_{i\in \mathrm{index}\left(LH\right)}\frac{Ex_{i}}{\left|lh\left(s_{i}\right)\right|}\left(\sum_{r\in lh\left(s_{i}\right)}r\right)\right),\\ En_{LH}=\sqrt{\frac{1}{\left|\mathrm{index}\left(LH\right)\right|}\left(\sum_{i\in \mathrm{index}\left(LH\right)}{\left(En_{i}\right)}^{2}\right)},\\ He_{LH}=\sqrt{\frac{1}{\left|\mathrm{index}\left(LH\right)\right|}\left(\sum_{i\in \mathrm{index}\left(LH\right)}{\left(He_{i}\right)}^{2}\right)}, \end{array}$$

where $\left|lh\left(s_{i}\right)\right|$ is the count of real numbers in $lh\left(s_{i}\right)$ and |index(LH)| is the cardinality of index(LH) defined as $\mathrm{index}\left(LH\right)=\left\{i|\left(s_{i},lh\left(s_{i}\right)\right)\in LH,lh\left(s_{i}\right)\neq \left\{0\right\}\right\}$ with $s_{i}\in S$.

## 4. The Proposed Robot Selection Approach

**Figure 1:**
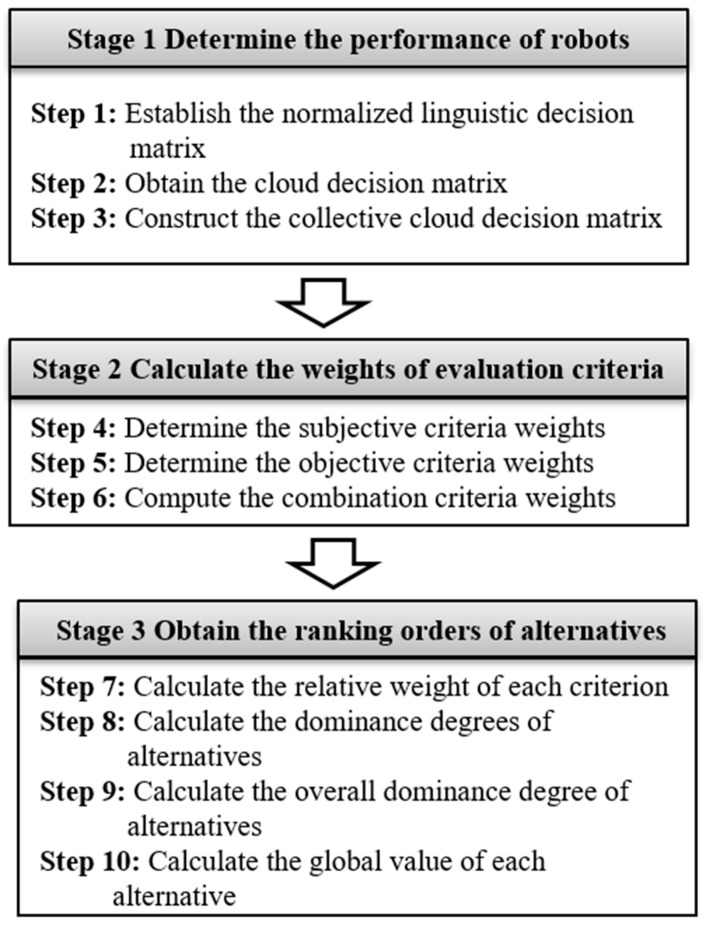
The proposed approach for robot selection.

This section develops a novel decision supporting method by combining cloud model with a modified TODIM to cope with the robot evaluation and selection problems considering subjective and objective criteria weights. In a nutshell, the new model comprises three key phases of determining the ratings for available robots, estimating the weights regarding evaluation criteria, and determining the ranking for alternative robots. First, the linguistic ratings of decision makers on alternatives are handled with the cloud model theory. Second, the importance weights of selection criteria are acquired by using a combination weighting method. Finally, the ranking orders of alternatives are determined adopting the procedure of a modified TODIM method. The overall process for using the proposed three-stage cloud TODIM model is shown in Figure 1, and the corresponding procedural steps are explained in the following subsections.

### 4.1. Determine Robot Assessments

Let us consider a robot selection problem with *m* possible alternatives $\left(A_{i},i=1,2,\ldots ,m\right)$ and *n* selection criteria $\left(C_{j},j=1,2,\ldots ,n\right)$, in which a collection of *l* experts or decision makers $\left({\mathrm{DM}}_{k},k=1,2,\ldots ,l\right)$ is involved. Let $D_{k}={\left[d_{ij}^{k}\right]}_{m\times n}$ be the linguistic decision matrix specified by DM*_k_*, where $d_{ij}^{k}$ denotes the judgement of alternative A*_i_* against C*_j_* assigned by ${\mathrm{DM}}_{k}$. Because decision makers from different working backgrounds have dissimilar experience and knowledge, they are given different weights $\lambda_{k}(k=1,2,\ldots ,l$ with $\sum_{k=1}^{l}\lambda_{k}=1$) in the robot selection process. Next, the cloud model is implemented to address the decision makers’ linguistic assessments of robots against each criterion.

**Step 1:** Establish the normalized linguistic decision matrix $R^{k}={\left(r_{ij}^{k}\right)}_{m\times n}$

In the real robot selection problem, different types of criteria often exist, such as cost and benefit. Therefore, we normalize the linguistic decision matrix $D^{k}={\left(d_{ij}^{k}\right)}_{m\times n}$ first to obtain the corresponding matrix $R^{k}={\left(r_{ij}^{k}\right)}_{m\times n}$, which can be yielded by

(6) $$r_{ij}^{k}=\{\begin{array}{c} d_{ij}^{k},\;\;\;\;\;\;\;\;\mathrm{for}\;\mathrm{benefit}\;\mathrm{critera}\\ neg\left(d_{ij}^{k}\right),\;\;\;\mathrm{for}\;\cos t\;\mathrm{criteria} \end{array}.$$

Note that if $d_{ij}^{k}$ is an LHFS $d_{ij}^{k}=\left\{s_{i},lh\left(s_{i}\right)\right\}$, then $r_{ij}^{k}=\left\{neg\left(s_{i}\right),lh\left(s_{i}\right)\right\}$.

**Step 2:** Obtain the cloud decision matrix ${\tilde{Y}}_{k}$

According to the introduced cloud conversion method in Section 3.2, this step is to convert the linguistic assessments of each normalized linguistic decision matrix $R^{k}$ into the corresponding normal clouds for determining the cloud decision matrix ${\tilde{Y}}_{k}={\left[{\tilde{y}}_{ij}^{k}\right]}_{m\times n}$, where ${\tilde{y}}_{ij}=\left(Ex_{ij}^{k},En_{ij}^{k},He_{ij}^{k}\right)\;\mathrm{for}\;i=1,2,\ldots ,m$ and j=1,2,…,n.

**Step 3:** Construct the collective cloud decision matrix $\tilde{Y}$

Once the decision makers’ cloud assessments are obtained, we can pull all individual cloud decision matrices ${\tilde{Y}}_{k}\left(k=1,2,\ldots ,l\right)$ for the collective cloud decision matrix $\tilde{Y}={\left[{\tilde{y}}_{ij}^{}\right]}_{m\times n}$. By using the CHA operator, the aggregated cloud rating of alternative *A_i_* on criterion C*_j_*, i.e., ${\tilde{y}}_{ij}^{}$, is calculated by

(7) $${\tilde{y}}_{ij}={\mathrm{CHA}}_{\omega ,\lambda }\left({\tilde{y}}_{ij}^{1},{\tilde{y}}_{ij}^{2},\ldots ,{\tilde{y}}_{ij}^{l}\right)=\sum_{h=1}^{l}\omega_{h}{\dot{\tilde{y}}}_{\sigma \left(h\right)},$$

where ${\dot{\tilde{y}}}_{ij}^{\sigma \left(h\right)}$ is the *h*th largest element of the weighted normal clouds ${\dot{\tilde{y}}}_{ij}^{k}\left({\dot{\tilde{y}}}_{ij}^{k}=l\lambda_{i}{\tilde{y}}_{ij}^{k},k=1,2,\ldots ,l\right)$ and $\omega =\left(\omega_{1},\omega_{2},\ldots ,\omega_{l}\right)$ is a related COWA weight vector with $\omega_{h}\in \left[0,1\right]$ and $\sum_{h=1}^{l}\omega_{h}=1$.

It is worth noting that if quantitative criteria existed in the problem of robot selection, the assessments of each alternative can be normalized, e.g., by using the linear normalization method [18], and converted into normal clouds. Thus, both qualitative and quantitative criteria can be handled in the course of robot evaluation and selection.

### 4.2. Determine Criteria Weights

Different types of weighting methods have been used to define the importance values of criteria for multi-criteria decision analysis, which can be categorized as subjective, objective, and combination weighting methods. The subjective weighting techniques, such as AHP [19], step-wise weight assessment ratio analysis (SWARA) [49,50], pivot pairwise relative criteria importance assessment (PIPRECIA) [51], factor relationship (FARE) [52], and best-worse method [53], assign the criteria weights using the subjective preferences or judgments of decision makers. In contrast, the objective weighting techniques, like entropy method [54], criterion impact loss (CILOS) [55], and integrated determination of objective criteria weights (IDOCRIW) [56,57], specify the criteria weights through dispersion analyses of data given in a decision matrix. Nevertheless, both the subjective and objective weighting techniques have their own advantages and drawbacks. Accordingly, the combination weighting methods integrating both subjective and objective weights have been increasingly adopted by researchers for deriving the weights of criteria.

In the sequel, an entropy-based combination weighting method is proposed to determine criteria weights for the robot selection problem.

**Step 4:** Determine the subjective criteria weights

Let $w_{j}^{k}$ be the linguistic weighting of criterion C*_j_* given by decision maker DM*_k_* to indicate its importance in the ranking of robots. The corresponding cloud weights ${\tilde{w}}_{j}^{k}\left(j=1,2,\ldots ,n\right)$ are aggregated to find the collective cloud weights $\tilde{w}={\left({\tilde{w}}_{j}\right)}_{1\times n}$ by using the CHA operator. Then, the subjective weight of each evaluation criterion is computed by

(8) $$w_{j}^{S}=\frac{\hat{s}\left({\tilde{w}}_{j}\right)}{\sum_{j=1}^{n}\hat{s}\left({\tilde{w}}_{j}\right)},$$

where $\hat{s}\left({\tilde{w}}_{j}\right)$ is the estimated value of the cloud weight ${\tilde{w}}_{j}$.

**Step 5:** Compute the objective criteria weights

On the basis of the entropy theory [54], the objective weights of criteria are defined as

(9) $$w_{j}^{O}=\frac{1-E_{j}}{n-\sum_{j=1}^{n}E_{j}},$$

where *E_j_* is the entropy of the projected results of the criterion C*_j_*, which can be obtained by

(10) $$E_{j}=-\left(\frac{1}{\ln m}\right)\sum_{i=1}^{m}P_{ij}\ln P_{ij},$$

(11) $$P_{ij}=\frac{\hat{s}\left({\tilde{y}}_{ij}\right)}{\sum_{i=1}^{m}\hat{s}\left({\tilde{y}}_{ij}\right)}.$$

**Step 6:** Compute the combination criteria weights

Based on the obtained subjective and objective weights, the combination weights of the criteria are computed by Equation (12) i.e.,

(12) $$w_{j}=\frac{\sqrt{w_{j}^{S}\cdot w_{j}^{O}}}{\sum_{j=1}^{n}\sqrt{w_{j}^{S}\cdot w_{j}^{O}}}.$$

### 4.3. Define the Ranking of Robots

The TODIM is an interactive MCDM method based on prospect theory [20,58], which reflects decision makers’ psychological behavior in the decision making process. It is a practical and consistent method to better make decisions and has been implemented successfully in many situations [22,25,27]. This subsection extends the TODIM method to cloud environment to help decision makers to rank alternative robots for the given robot selection problem.

**Step 7:** Compute the relative weight of C*_j_* with respect to the reference criterion C*_r_* by

(13) $$w_{jr}=\frac{w_{j}}{w_{r}},\;\;\;j=1,2,\ldots ,n,$$

where $w_{r}=\underset{1\leq j\leq n}{\max}\left\{w_{j}\right\}$ and C*_r_* is the criterion associated with *w_r_*.

**Step 8:** Determine the domination degree of *A_i_* over *A_p_* under C*_j_*, i.e.,

(14) $$\begin{array}{l} \varphi_{j}\left(A_{i},A_{p}\right)=\{\begin{array}{c} \sqrt{d\left({\tilde{y}}_{ij},{\tilde{y}}_{pj}\right)w_{jr}/\sum_{j=1}^{n}w_{jr}},\;\;\;\;\;\;\;\mathrm{if}\;{\tilde{y}}_{ij}>{\tilde{y}}_{pj}\\ 0,\;\;\;\;\;\;\;\;\;\;\;\;\;\;\;\;\;\;\;\;\;\;\;\;\;\;\;\;\;\;\;\;\;\;\;\;\;\;\;\;\mathrm{if}\;{\tilde{y}}_{ij}={\tilde{y}}_{pj}\\ -\frac{1}{\theta }\sqrt{d\left({\tilde{y}}_{ij},{\tilde{y}}_{pj}\right)\sum_{j=1}^{n}w_{jr}/w_{jr}},\;\;\;\mathrm{if}\;{\tilde{y}}_{ij}<{\tilde{y}}_{pj} \end{array},\;\;\;\\ \;\;\;\;\;\;\;\;\;\;\;\;\;\;\;\;\;\;\;\;\;\;i,p=1,2,\ldots ,m,j=1,2,\ldots ,n, \end{array}$$

where *θ* is the attenuation factor of the losses and $d\left({\tilde{y}}_{ij},{\tilde{y}}_{pj}\right)$ is the distance between the cloud ratings ${\tilde{y}}_{ij}$ and ${\tilde{y}}_{pj}$.

**Step 9:** Obtain the overall domination degree of *A_i_* over *A_p_* by

(15) $$\varphi \left(A_{i},A_{p}\right)=\sum_{j=1}^{n}\varphi_{j}\left(A_{i},A_{p}\right),\;\;\;i,p=1,2,\ldots ,m.$$

**Step 10:** Acquire the global value of alternative *A_i_* over the other alternatives by using the following equation:

(16) $$\xi \left(A_{i}\right)=\frac{\delta \left(A_{i}\right)-\underset{1\leq i\leq m}{\min}\left\{\delta \left(A_{i}\right)\right\}}{\underset{1\leq i\leq m}{\max}\left\{\delta \left(A_{i}\right)\right\}-\underset{1\leq i\leq m}{\min}\left\{\delta \left(A_{i}\right)\right\}},\;\;\;i=1,2,\ldots ,m,$$

where $\delta \left(A_{i}\right)=\sum_{p=1}^{m}\varphi \left(A_{i},A_{p}\right)$. All the alternatives are ranked following the descending order of their global values $\xi \left(A_{i}\right)\left(i=1,2,\ldots ,m\right)$, and the best robot can be derived for the considered industrial application.

## 5. Case Study

### 5.1. Application

This section presents an empirical case adapted from [6] to account for the applicability and potential of our proposed robot selection approach. An automobile manufacturing company wants to choose a robot for implementing its manufacturing process. After preliminary selection, four candidates referred as *A*_1_, *A*_2_, *A*_3_, and *A*_4_ are left for further assessment. Besides, seven evaluation criteria are considered for selecting the most appropriate robot: Inconsistency with infrastructure (C_1_), Man-machine interface (C_2_), Programming flexibility (C_3_), Vendor’s service contract (C_4_), Supporting channel partner’s performance (C_5_), Compliance (C_6_), and Stability (C_7_). All these criteria are benefit criteria except C_1_, which is a cost criterion.

An expert group which consists of five decision makers (DM_1_, DM_2_,…, DM_5_) is established for the evaluation and selection of the most suitable robot. The decision makers’ weights are set as 0.20, 0.30, 0.10, 0.25, and 0.15, respectively, due to their differentiated knowledge and backgrounds. According to the materials and data concerning the considered robots, a seven-point linguistic term set *S* is adopted by the experts to evaluate the given robots and the criteria importance weights, i.e.,

$$S=\left\{\begin{array}{l} s_{0}=\mathrm{Very}\;\mathrm{Low}\;(\mathrm{VL}),s_{1}=\;\mathrm{Low}\;(L),s_{2}=\;\mathrm{Medium}\mathrm{Low}\;(\mathrm{ML}),s_{3}=\;\mathrm{Medium}\;(M),\\ s_{4}=\;\mathrm{Medium}\;\mathrm{High}\;(\mathrm{MH}),s_{5}=\;\mathrm{High}\;(H),s_{6}=\;\mathrm{Very}\;\mathrm{High}\;(\mathrm{VH}) \end{array}\right\}.$$

As a result, the performance ratings of the four alternative robots against each criterion and the relative prominence of the seven criteria are indicated in Table 1 and Table 2, respectively.

By applying the proposed cloud TODIM approach, the above robot selecting problem is solved and the procedural steps are summarized as follows.

**Stage 1.** Evaluate the performance of robots

Step 1: Since criterion C_1_ is cost type, the linguistic ratings of the alternatives about C_1_ are normalized and listed in Table 3 and the normalized linguistic decision matrix $R^{k}={\left(r_{ij}^{k}\right)}_{4\times 7}\left(k=1,2,\ldots ,5\right)$ can be constructed accordingly.

Step 2: By using the cloud conversion method, we can obtain the cloud decision matrixes ${\tilde{Y}}_{k}={\left[{\tilde{y}}_{ij}^{k}\right]}_{4\times 7}$ (k=1,2,…,5) as tabulated in Table 4. It is assumed that the valid universe is [0, 10] and *He*_3_ = 0.1 in the computation.

Step 3: With Table 4 and Equation (7), the collective cloud decision matrix $\tilde{Y}={\left[{\tilde{y}}_{ij}\right]}_{4\times 7}$ is built as shown in Table 5. It may be mentioned here that the COWA weight vector is computed as ω=(0.112,0.236,0.304,0.236,0.112) by using the normal distribution-based method [45].

**Stage 2.** Calculate the criteria weights

Step 4: Based on Table 2 and the CHA operator, the collective cloud weight vector $\tilde{w}={\left({\tilde{w}}_{j}\right)}_{1\times 7}$ is computed as shown in the last row of Table 5. By Equation (8), we can derive the subjective criteria weights: $w_{1}^{S}=0.213,w_{2}^{S}=0.126,w_{3}^{S}=0.139,w_{4}^{S}=0.204,w_{5}^{S}=0.157,w_{6}^{S}=0.160,andw_{7}^{S}=0.154$.

Step 5: According to the entropy method, the values of *P_ij_* and *E_j_* are derived by Equations (10) and (11), which are furnished in Table 6, and the objective criteria weights are determined via Equation (9) as: $w_{1}^{O}=0.435,w_{2}^{O}=0.147,w_{3}^{O}=0.082,w_{4}^{O}=0.083,w_{5}^{O}=0.078,w_{6}^{O}=0.065,w_{7}^{O}=0.109$.

Step 6: Using the subjective and objective criteria weights, Equation (12) is used to compute the combination criteria weights shown as follows:

$$w_{1}=0.299,w_{2}=0.133,w_{3}=0.104,w_{4}=0.128,w_{5}=0.109,w_{6}=0.100,\mathrm{and}w_{7}=0.127.$$

**Stage 3.** Acquire the ranking orders of alternatives

Step 7: The relative weights of evaluation criteria regarding the reference criterion C*_r_* can be calculated through Equation (13) as:

$$w_{1r}=1.00,w_{2r}=0.446,w_{3r}=0.350,w_{4r}=0.427,w_{5r}=0.364,w_{6r}=0.335,w_{7r}=0.426.$$

Step 8: Based on Table 5, we use Equation (14) to obtain the dominance degrees between the alternatives with respect to the seven criteria. Without loss of generality, the attenuation coefficient *θ* is set to be 1. The above results are indicated in Table 7.

Steps 9 and 10: The overall dominance degree for each pair of alternatives on all the criteria is computed by Equation (15), and the global values of the alternative robots are determined using Equation (16). We summarized the results of these steps in Table 8. It can be observed that alternative *A*_1_ with the top global value is the best robot for the considered case study, and the ranking of the four robots is $A_{1}≻A_{4}≻A_{3}≻A_{2}.$

### 5.2. Sensitivity Analysis

In order to investigate the influence of the weight values of criteria on the ranking orders of alternatives, a sensitivity analysis about criteria weights is conducted. Four cases are considered in the analysis: combination weights (Case 1), subjective weights (Case 2), objective weights (Case 3), and equal weights (Case 4). Table 9 shows the global values of alternatives with different sets of criteria weights. From Table 9, it is apparent that the change of weight values leads to the change of global values of the alternatives. Depending on different weight values of criteria, the ranking orders of alternatives may be different, and the results may lead to different decisions. So, it is important to choose an appropriate method for determining criteria weights. The entropy-based combination weighting method proposed in this paper can generate more reliable weight values of criteria since both subjective and objective weights are taken into account. In addition, the final ranking of the alternative robots is $A_{1}≻A_{4}≻A_{3}≻A_{2}$ in the four cases, which is stable under the changing weight values of criteria. This shows that the ranking result obtained by the proposed cloud TODIM approach is reasonable and robust for the given case study.

### 5.3. Comparison Analysis

**Figure 2:**
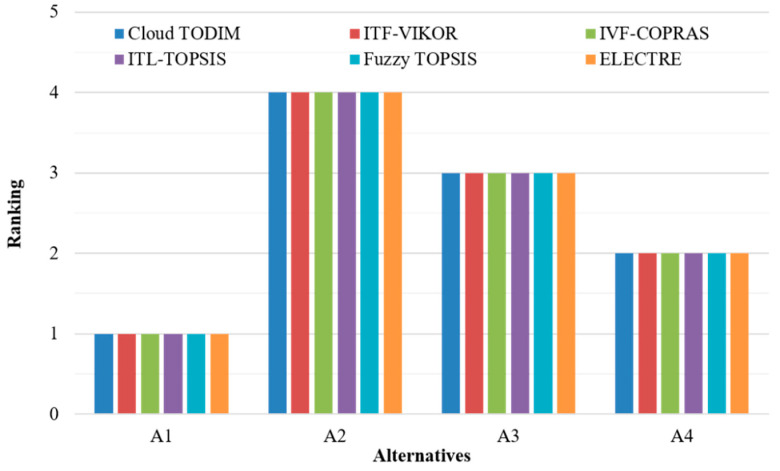
Ranking comparison.

For illustrating the efficiency of the suggested decision support framework, we compared with other representative methods for the same robot selection case, which include the interval type-2 fuzzy VIKOR (ITF-VIKOR) [6], the interval-valued fuzzy COPRAS (IVF-COPRAS) [31], the interval 2-tuple linguistic TOPSIS (ITL-TOPSIS) [18], the fuzzy TOPSIS [5], and the ELECTRE [2] methods. The order results for the given robots with these five approaches are shown in Figure 2.

From Figure 2, we can see that the sequences of alternatives obtained by the listed methods and the proposed cloud TODIM model are exactly the same, and the best-suited robot is alternative *A*_1_. This shows the verification and validation of the proposed approach. However, compared with other robot selection methods, the presented cloud TODIM model has the following merits:

1. The performance ratings of robots are evaluated in linguistic expressions and the hesitancy and inconsistency in the decision makers’ evaluations on robots can be well represented. This allows decision makers to define their opinions more realistically and make the robot selection easier to perform.
2. Based on the cloud model, the new approach can not only reflect average level but also the vagueness and randomness of the evaluation criteria. Moreover, the aggregation of performance information utilizing the CHA operator can reflect the importance weights of experts and simultaneously minimize the effect of biased assessments on the ranking results.
3. We consider both subjective and objective weights of criteria in ranking the alternative robots, and the combination criteria weights are computed directly without the need to determine the weight coefficient between subjective and objective weights in advance. This makes the ranking results more accurate and theoretically reasonable.
4. By applying an extended TODIM method, the presented approach takes the behavioral characteristics of decision makers (e.g., reference dependence and loss aversion) into consideration in determining the ranking of robots. Therefore, the robot selection approach proposed in this paper is more realistic in practical applications.

## 6. Conclusions

Industrial robots are commonly applied in different advanced manufacturing systems to enhance efficiency and improve product quality. Choosing the ideal robot for a particular problem has nowadays become a major concern for manufacturing companies. This work developed an integrated MCDM approach combing the cloud model and TODIM method for the selection of the optimal industrial robot. We used an illustrative example for indicating the applicability and suitability of our proposed model. To validate the results, the ranking results derived with the cloud TODIM approach were compared with some existing robot selection methods. The results display that the proposed model is more powerful in dealing with the uncertainty and imprecision of subjective assessments given by decision makers. It can generate a rational ranking result for the given robots in a real-life robot selection problem and accommodate situations in which decision makers show bounded rationality. In addition, the proposed robot section model sufficiently considers the different importance of evaluation criteria, which makes the ranking orders more consistent with the actual situation.

For future research, the following directions are recommended. First, the interrelationships among criteria were ignored in this study. To overcome this deficiency, the proposed approach can be improved by using the fuzzy cognitive map or decision-making trial and evaluation laboratory (DEMATEL) method. Second, many computations are involved in the proposed approach and it requires additional expertise for practitioners in the adopted methods and cloud model theory. Hence, our future research will develop a specialized software tool for the proposed robot selection approach so that it can be easily implemented by non-experts. In addition, the proposed cloud TODIM framework for evaluating and selecting robots is a general method, which is able to be applied to other manufacturing problems for making a suitable decision, including rapid prototyping process selection, flexible manufacturing system selection, and advanced manufacturing technology selection.

## Figures and Tables

**Table 1:** Linguistic evaluations of alternative robots.

Robots	Experts	Criteria						
		C_1_	C_2_	C_3_	C_4_	C_5_	C_6_	C_7_
*A*_1_	DM_1_	*s*_0_	{(*s*_6_, 0.6, 0.9)}	*s*_3_	*s*_4_	*s*_4_	*s*_6_	*s*_6_
	DM_2_	{(*s*_0_, 0.7)}	*s*_6_	*s*_4_	*s*_4_	*s*_5_	*s*_5_	*s*_5_
	DM_3_	*s*_1_	*s*_5_	*s*_3_	*s*_4_	*s*_4_	{(*s*_5_, *s*_6_)}	*s*_6_
	DM_4_	*s*_1_	*s*_6_	*s*_3_	*s*_5_	*s*_4_	*s*_5_	*s*_6_
	DM_5_	*s*_1_	*s*_5_	{(*s*_3_, 0.8), (*s*_4_, 0.9)}	*s*_5_	{(*s*_5_, 0.6, 0.8)}	*s*_6_	{(*s*_5_, 0.7), (*s*_6_, 0.6, 0.9)}
*A*_2_	DM_1_	*s*_6_	*s*_1_	*s*_2_	*s*_3_	*s*_5_	*s*_4_	{(*s*_3_, 0.7, 0.9)}
	DM_2_	*s*_6_	*s*_2_	*s*_1_	*s*_2_	*s*_5_	*s*_3_	*s*_2_
	DM_3_	{(*s*_5_, *s*_6_)}	{(*s*_1_, 0.6, 0.8)}	*s*_1_	{(*s*_3_, 0.6)}	*s*_4_	*s*_3_	*s*_3_
	DM_4_	{(*s*_5_, 0.5, 0.8)}	*s*_2_	{(*s*_2_, 0.8)}	*s*_2_	*s*_4_	*s*_5_	*s*_3_
	DM_5_	*s*_5_	*s*_2_	*s*_2_	*s*_3_	*s*_5_	*s*_4_	*s*_2_
*A*_3_	DM_1_	*s*_2_	*s*_3_	*s*_5_	*s*_5_	*s*_3_	{(*s*_4_, 0.5, 0.7)}	*s*_3_
	DM_2_	*s*_1_	*s*_2_	*s*_4_	*s*_4_	*s*_2_	*s*_3_	*s*_2_
	DM_3_	*s*_2_	{(*s*_3_, 0.8)}	{(*s*_5_, 0.7)}	*s*_5_	*s*_3_	*s*_4_	*s*_2_
	DM_4_	*s*_2_	*s*_3_	*s*_5_	*s*_5_	*s*_2_	*s*_3_	*s*_3_
	DM_5_	{(*s*_0_, 0.6, 0.8), (*s*_1_, 0.7)}	{(*s*_4_, 0.3, 0.5, 0.8)}	*s*_4_	*s*_4_	{(*s*_3_, 0.7), (*s*_4_, 0.6)}	*s*_4_	*s*_3_
*A*_4_	DM_1_	*s*_1_	*s*_4_	*s*_4_	*s*_3_	*s*_6_	*s*_5_	*s*_5_
	DM_2_	*s*_0_	*s*_5_	*s*_5_	*s*_2_	*s*_6_	*s*_5_	*s*_6_
	DM_3_	*s*_1_	*s*_4_	{(*s*_4_, *s*_5_)}	{(*s*_2_, 0.4), (*s*_3_, 0.7), (*s*_4_, 0.4)}	*s*_6_	*s*_4_	{(*s*_6_, 0.6)}
	DM_4_	*s*_1_	*s*_4_	*s*_4_	*s*_1_	*s*_5_	*s*_4_	*s*_5_
	DM_5_	{(*s*_0_, 0.7, 0.8)}	*s*_5_	*s*_5_	*s*_2_	*s*_5_	*s*_5_	*s*_5_

**Table 2:** Linguistic evaluations of criteria weights.

Experts	Criteria						
	C_1_	C_2_	C_3_	C_4_	C_5_	C_6_	C_7_
DM_1_	*s*_5_	*s*_3_	*s*_5_	*s*_4_	*s*_5_	*s*_4_	*s*_5_
DM_2_	*s*_6_	*s*_3_	*s*_4_	*s*_6_	*s*_4_	*s*_5_	*s*_4_
DM_3_	*s*_5_	*s*_4_	*s*_4_	*s*_4_	*s*_4_	*s*_5_	*s*_5_
DM_4_	*s*_6_	*s*_4_	*s*_3_	*s*_6_	*s*_4_	*s*_5_	*s*_4_
DM_5_	*s*_6_	*s*_3_	*s*_3_	*s*_6_	*s*_5_	*s*_4_	*s*_4_

**Table 3:** Normalized linguistic assessments of alternatives for criterion C_1_.

Experts	Alternatives			
	*A*_1_	*A*_2_	*A*_3_	*A*_4_
DM_1_	*S*_6_	*s*_0_	*s*_4_	*s*_5_
DM_2_	{(*s*_6_, 0.7)}	*s*_0_	*s*_5_	*s*_6_
DM_3_	*s*_5_	{(*s*_0_, *s*_1_)}	*s*_4_	*s*_5_
DM_4_	*s*_5_	{(*s*_1_, 0.5, 0.8)}	*s*_4_	*s*_5_
DM_5_	*s*_5_	*s*_1_	{(*s*_5_, 0.7), (*s*_6_, 0.6, 0.8)}	{(*s*_6_ 0.7, 0.8)}

**Table 4:** Cloud decision matrix ${\tilde{Y}}_{k}$ .

Alternatives	Experts	Criteria						
		C_1_	C_2_	C_3_	C_4_	C_5_	C_6_	C_7_
*A*_1_	DM_1_	(10, 0.833, 0.424)	(7.5, 0.833, 0.424)	(5, 0.197, 0.1)	(5.96, 0.318, 0.162)	(5.96, 0.318, 0.162)	(10, 0.833, 0.424)	(10, 0.833, 0.424)
	DM_2_	(7, 0.833, 0.424)	(10, 0.833, 0.424)	(5.96, 0.318, 0.162)	(5.96, 0.318, 0.162)	(7.5, 0.515, 0.262)	(7.5, 0.515, 0.262)	(7.5, 0.515, 0.262)
	DM_3_	(7.5, 0.515, 0.262)	(7.5, 0.515, 0.262)	(5, 0.197, 0.1)	(5.96, 0.318, 0.162)	(5.96, 0.318, 0.162)	(8.75, 0.693, 0.352)	(10, 0.833, 0.424)
	DM_4_	(7.5, 0.515, 0.262)	(10, 0.833, 0.424)	(5, 0.197, 0.1)	(7.5, 0.515, 0.262)	(5.96, 0.318, 0.162)	(7.5, 0.515, 0.262)	(10, 0.833, 0.424)
	DM_5_	(7.5, 0.515, 0.262)	(7.5, 0.515, 0.262)	(4.68, 0.265, 0.135)	(7.5, 0.515, 0.262)	(5.25, 0.515, 0.262)	(10, 0.833, 0.424)	(6.38, 0.693, 0.352)
*A*_2_	DM_1_	(0, 0.833, 0.424)	(2.5, 0.515, 0.262)	(4.05, 0.318, 0.162)	(5, 0.197, 0.1)	(7.5, 0.515, 0.262)	(5.96, 0.318, 0.162)	(4, 0.197, 0.1)
	DM_2_	(0, 0.833, 0.424)	(4.05, 0.318, 0.162)	(2.5, 0.515, 0.262)	(4.05, 0.318, 0.162)	(7.5, 0.515, 0.262)	(5, 0.197, 0.1)	(4.05, 0.318, 0.162)
	DM_3_	(1.25, 0.693, 0.352)	(1.75, 0.515, 0.262)	(2.5, 0.515, 0.262)	(3, 0.197, 0.1)	(5.96, 0.318, 0.162)	(5, 0.197, 0.1)	(5, 0.197, 0.1)
	DM_4_	(1.63, 0.515, 0.262)	(4.05, 0.318, 0.162)	(3.24, 0.318, 0.162)	(4.05, 0.318, 0.162)	(5.96, 0.318, 0.162)	(7.5, 0.515, 0.262)	(5, 0.197, 0.1)
	DM_5_	(2.5, 0.515, 0.262)	(4.05, 0.318, 0.162)	(4.05, 0.318, 0.162)	(5, 0.197, 0.1)	(7.5, 0.515, 0.262)	(5.96, 0.318, 0.162)	(4.05, 0.318, 0.162)
*A*_3_	DM_1_	(5.96, 0.318, 0.162)	(5, 0.197, 0.1)	(7.5, 0.515, 0.262)	(7.5, 0.515, 0.262)	(5, 0.197, 0.1)	(3.58, 0.318, 0.162)	(5, 0.197, 0.1)
	DM_2_	(7.5, 0.515, 0.262)	(4.05, 0.318, 0.162)	(5.96, 0.318, 0.162)	(5.96, 0.318, 0.162)	(4.05, 0.318, 0.162)	(5, 0.197, 0.1)	(4.05, 0.318, 0.162)
	DM_3_	(5.96, 0.318, 0.162)	(4, 0.197, 0.1)	(5.25, 0.515, 0.262)	(7.5, 0.515, 0.262)	(5, 0.197, 0.1)	(5.96, 0.318, 0.162)	(4.05, 0.318, 0.162)
	DM_4_	(5.96, 0.318, 0.162)	(5, 0.197, 0.1)	(7.5, 0.515, 0.262)	(7.5, 0.515, 0.262)	(4.05, 0.318, 0.162)	(5, 0.197, 0.1)	(5, 0.197, 0.1)
	DM_5_	(6.13, 0.693, 0.352)	(3.18, 0.318, 0.162)	(5.96, 0.318, 0.162)	(5.96, 0.318, 0.162)	(3.54, 0.265, 0.135)	(5.96, 0.318, 0.162)	(5, 0.197, 0.1)
*A*_4_	DM_1_	(7.5, 0.515, 0.262)	(5.96, 0.318, 0.162)	(5.96, 0.318, 0.162)	(5, 0.197, 0.1)	(10, 0.833, 0.424)	(7.5, 0.515, 0.262)	(7.5, 0.515, 0.262)
	DM_2_	(10, 0.833, 0.424)	(7.5, 0.515, 0.262)	(7.5, 0.515, 0.262)	(4.05, 0.318, 0.162)	(10, 0.833, 0.424)	(7.5, 0.515, 0.262)	(10, 0.833, 0.424)
	DM_3_	(7.5, 0.515, 0.262)	(5.96, 0.318, 0.162)	(6.73, 0.428, 0.218)	(2.5, 0.283, 0.144)	(10, 0.833, 0.424)	(5.96, 0.318, 0.162)	(6, 0.833, 0.424)
	DM_4_	(7.5, 0.515, 0.262)	(5.96, 0.318, 0.162)	(5.96, 0.318, 0.162)	(2.5, 0.515, 0.262)	(7.5, 0.515, 0.262)	(5.96, 0.318, 0.162)	(7.5, 0.515, 0.262)
	DM_5_	(7.5, 0.833, 0.424)	(7.5, 0.515, 0.262)	(7.5, 0.515, 0.262)	(4.05, 0.318, 0.162)	(7.5, 0.515, 0.262)	(7.5, 0.515, 0.262)	(7.5, 0.515, 0.262)

**Table 5:** Collective cloud decision matrix $\tilde{Y}$ and Collective cloud weight vector $\tilde{w}$.

Alternatives	Criteria						
	C_1_	C_2_	C_3_	C_4_	C_5_	C_6_	C_7_
*A*_1_	(8.13, 0.665, 0.339)	(8.66, 0.771, 0.392)	(5.1, 0.234, 0.119)	(6.63, 0.395, 0.201)	(6.09, 0.397, 0.202)	(8.73, 0.678, 0.345)	(8.81, 0.724, 0.368)
*A*_2_	(0.86, 0.661, 0.337)	(3.49, 0.384, 0.195)	(3.41, 0.441, 0.225)	(4.45, 0.260, 0.132)	(6.96, 0.445, 0.227)	(5.97, 0.319, 0.162)	(4.35, 0.271, 0.138)
*A*_3_	(6.25, 0.442, 0.225)	(4.44, 0.271, 0.138)	(6.79, 0.431, 0.220)	(6.91, 0.431, 0.220)	(4.30, 0.271, 0.138)	(4.78, 0.253, 0.129)	(4.77, 0.256, 0.130)
*A*_4_	(7.92, 0.643, 0.327)	(6.49, 0.397, 0.202)	(6.51, 0.411, 0.209)	(3.67, 0.386, 0.197)	(8.78, 0.683, 0.348)	(7.68, 0.445, 0.226)	(7.84, 0.601, 0.306)
$\tilde{w}$	(7.92, 0.643, 0.327)	(6.49, 0.397, 0.202)	(6.51, 0.411, 0.209)	(3.67, 0.386, 0.197)	(8.78, 0.683, 0.348)	(7.68, 0.445, 0.226)	(7.84, 0.601, 0.306)

**Table 6:** Values of *P_ij_* and *E_j_* by the entropy method.

P_ij_	C_1_	C_2_	C_3_	C_4_	C_5_	C_6_	C_7_
*A*_1_	0.350	0.375	0.234	0.305	0.233	0.323	0.342
*A*_2_	0.037	0.151	0.155	0.205	0.266	0.220	0.169
*A*_3_	0.271	0.192	0.313	0.320	0.164	0.175	0.186
*A*_4_	0.341	0.282	0.297	0.170	0.337	0.283	0.303
*E_j_*	0.874	0.957	0.976	0.976	0.977	0.981	0.968

**Table 7:** Dominance degrees between alternatives under each criterion.

	C_1_	C_2_	C_3_	C_4_	C_5_	C_6_	C_7_
$\varphi_{j}\left(A_{1},A_{2}\right)$	1.038	0.378	0.581	0.375	−0.184	0.575	0.590
$\varphi_{j}\left(A_{1},A_{3}\right)$	0.741	0.632	−0.501	−0.240	0.331	0.559	0.644
$\varphi_{j}\left(A_{1},A_{4}\right)$	0.227	0.664	−0.486	0.417	−0.504	0.524	0.365
$\varphi_{j}\left(A_{2},A_{3}\right)$	−1.100	−0.491	−0.434	−0.426	0.371	0.248	−0.242
$\varphi_{j}\left(A_{2},A_{4}\right)$	−1.045	−0.421	−0.446	0.494	−0.496	−0.365	−0.540
$\varphi_{j}\left(A_{3},A_{4}\right)$	−0.711	−0.360	0.139	0.364	−0.524	−0.415	−0.600
$\varphi_{j}\left(A_{2},A_{1}\right)$	−1.038	−0.378	−0.581	−0.375	0.184	−0.575	−0.590
$\varphi_{j}\left(A_{3},A_{1}\right)$	−0.741	−0.632	0.501	0.240	−0.331	−0.559	−0.644
$\varphi_{j}\left(A_{4},A_{1}\right)$	−0.227	−0.664	0.486	−0.417	0.504	−0.524	−0.365
$\varphi_{j}\left(A_{3},A_{2}\right)$	1.100	0.491	0.434	0.426	−0.371	−0.248	0.242
$\varphi_{j}\left(A_{4},A_{2}\right)$	1.045	0.421	0.446	−0.494	0.496	0.365	0.540
$\varphi_{j}\left(A_{4},A_{3}\right)$	0.711	0.360	−0.139	−0.364	0.524	0.415	0.600

**Table 8:** Overall dominance degrees between alternatives and global value of each alternative.

$\varphi \left(A_{1},A_{2}\right)$	$\varphi \left(A_{1},A_{3}\right)$	$\varphi \left(A_{1},A_{4}\right)$	$\delta \left(A_{1}\right)$	$\xi \left(A_{i}\right)$
3.353	2.166	1.207	6.727	1.000
$\varphi \left(A_{2},A_{1}\right)$	$\varphi \left(A_{2},A_{3}\right)$	$\varphi \left(A_{2},A_{4}\right)$	$\delta \left(A_{2}\right)$	$\xi \left(A_{2}\right)$
−3.353	−2.073	−2.820	−8.246	0.000
$\varphi \left(A_{3},A_{1}\right)$	$\varphi \left(A_{3},A_{2}\right)$	$\varphi \left(A_{3},A_{4}\right)$	$\delta \left(A_{3}\right)$	$\xi \left(A_{3}\right)$
−2.166	2.073	−2.107	−2.200	0.404
$\varphi \left(A_{4},A_{1}\right)$	$\varphi \left(A_{4},A_{2}\right)$	$\varphi \left(A_{4},A_{3}\right)$	$\delta \left(A_{4}\right)$	$\xi \left(A_{4}\right)$
−1.207	2.820	2.107	3.720	0.799

**Table 9:** Results of sensitivity analysis.

Alternatives	Case 1	Case 2	Case 3	Case 4
*A*_1_	1.000	1.000	1.000	1.000
*A*_2_	0.000	0.000	0.000	0.000
*A*_3_	0.407	0.413	0.402	0.417
*A*_4_	0.832	0.837	0.824	0.825
